# Preoperative Central Sensitization as a Predictor of Pain Outcomes After Hip and Knee Arthroplasty: A Systematic Review

**DOI:** 10.7759/cureus.101791

**Published:** 2026-01-18

**Authors:** Majed S Alasbali, Yaser A Alshammari, Mohammed S Alhomaidi, Ibrahim A Alaqil, Murad A Sharif, Abdulelah M Al-Shahir, Abdullah J Al‎‎‎‎ghamdi, Abdulkarim O Althobaiti, Hetaf M Alotaibi, Ahmed M Elsayed

**Affiliations:** 1 Department of Orthopedic Surgery, King Fahad Medical City, Riyadh, SAU; 2 College of Medicine, Hail University, Hail, SAU; 3 College of Medicine, Jazan University, Jazan, SAU; 4 College of Medicine, King Khalid University, Abha, SAU; 5 College of Medicine, Alexandria University, Alexandria, EGY; 6 Faculty of Medicine, University of Jeddah, Jeddah, SAU; 7 Faculty of Medicine, King Abdulaziz University, Jeddah, SAU; 8 Department of Pediatric Rehabilitation, Physiobasic Rehabilitation Center, Jazan, SAU

**Keywords:** central sensitization, central sensitization inventory, chronic postsurgical pain, hip arthroplasty, knee arthroplasty, pain prediction, postoperative pain, quantitative sensory testing

## Abstract

Persistent pain remains a common clinical challenge after hip and knee arthroplasty, even when surgical outcomes are technically successful, with growing recognition that altered central pain processing, particularly central sensitization (CS), may contribute to unfavorable postoperative outcomes. This systematic review evaluated whether preoperative CS predicts poor pain-related outcomes after hip and knee arthroplasty. A comprehensive literature search was conducted in PubMed, Scopus, Web of Science, and the Cochrane Library from database inception to the final search date, identifying observational studies that assessed CS using validated instruments such as the Central Sensitization Inventory (CSI) and quantitative sensory testing (QST) in patients undergoing total knee arthroplasty (TKA), unicompartmental knee arthroplasty, revision knee arthroplasty, or total hip arthroplasty (THA). Extracted outcomes included postoperative pain intensity, functional outcomes, patient satisfaction, analgesic consumption, development of chronic postsurgical pain, and achievement of minimal clinically important differences (MCIDs), while methodological quality was evaluated using the Newcastle-Ottawa Scale (NOS). Nine observational studies met the inclusion criteria, and across these studies, preoperative CS was consistently associated with higher postoperative pain intensity, an increased risk of chronic postsurgical pain, a lower likelihood of achieving clinically meaningful pain improvement, greater use of analgesics, and reduced patient satisfaction. In contrast, patients without evidence of CS experienced superior pain relief and functional recovery across all follow-up periods, including short-, mid-, and long-term. Overall, study quality ranged from moderate to high. These findings indicate that preoperative CS is a strong predictor of poor pain-related outcomes following hip and knee arthroplasty, and incorporating CS assessment into preoperative evaluation may support improved risk stratification and the development of personalized perioperative pain management strategies to optimize postoperative outcomes.

## Introduction and background

Hip and knee arthroplasty are among the most effective surgical interventions for relieving pain and improving function in patients with advanced osteoarthritis [1,2]. Although most patients achieve substantial symptomatic improvement, a notable proportion continue to experience persistent or chronic pain after surgery despite technically successful procedures and the absence of mechanical complications [3,4]. Such ongoing pain negatively affects patient satisfaction and functional recovery and contributes to increased healthcare utilization and prolonged rehabilitation [5].

Postoperative pain after arthroplasty has traditionally been attributed to peripheral factors such as surgical trauma, inflammation, and residual joint pathology [6]. However, these factors do not fully explain the frequent mismatch between favorable structural outcomes and persistent patient-reported pain [4,7]. This discrepancy has led to increasing interest in centrally mediated pain mechanisms, particularly central sensitization (CS), as contributors to unfavorable postoperative pain outcomes [7,8].

CS is characterized by heightened excitability of the central nervous system, resulting in amplified pain responses to both noxious and non-noxious stimuli [8]. Clinically, it presents as widespread pain sensitivity, hyperalgesia, allodynia, and impaired pain modulation [8,9]. In patients with osteoarthritis, CS has been associated with greater pain severity, functional impairment, and reduced responsiveness to treatments targeting peripheral joint pathology, including surgical intervention [7-9].

CS can be assessed using validated patient-reported instruments, such as the Central Sensitization Inventory (CSI), as well as objective methods, including quantitative sensory testing (QST), which evaluates pain thresholds and pain modulation mechanisms [8,9]. These approaches allow stratification of patients according to pain processing characteristics and facilitate investigation of CS as a prognostic factor for surgical outcomes [7,9].

Although growing evidence suggests that CS is associated with poorer outcomes after arthroplasty, existing studies vary widely in surgical procedures, assessment tools, outcome measures, and follow-up durations [3,7]. Consequently, the independent predictive value of CS for postoperative pain outcomes remains incompletely defined [7,9]. This systematic review, therefore, aims to synthesize the available evidence on CS as a predictor of pain-related outcomes following hip and knee arthroplasty, focusing on pain intensity, persistence, functional recovery, and patient satisfaction [1-9].

## Review

Methodology

Literature Search Strategy

This systematic review was conducted in accordance with the Preferred Reporting Items for Systematic Reviews and Meta-Analyses (PRISMA) guidelines [10]. A comprehensive electronic search was performed in PubMed, Scopus, Web of Science, and the Cochrane Library from database inception to the final search date. The search combined controlled vocabulary and free-text terms related to CS and altered pain processing, arthroplasty procedures, and postoperative pain-related outcomes. Key terms included variations of CS, CSI, pain catastrophizing, and QST, combined with terms related to joint replacement, total knee arthroplasty (TKA), and total hip arthroplasty (THA). Searches were limited to human studies and original research articles. Reviews, editorials, conference abstracts without full data, study protocols, and non-relevant publications were excluded.

Eligibility Criteria

Eligibility criteria were defined a priori using a population-exposure-outcome framework. Included studies investigated adult patients undergoing primary or revision hip or knee arthroplasty, including THA, TKA, or unicompartmental knee arthroplasty (UKA). Studies were required to assess preoperative CS or centrally mediated pain mechanisms using validated instruments such as the CSI or QST and to report postoperative pain-related outcomes, including pain intensity, chronic postoperative pain, functional outcomes, quality of life, or patient satisfaction at a defined follow-up. Prospective and retrospective observational studies, as well as randomized controlled trials, were eligible. There were no restrictions on geographic location or clinical setting. Studies not involving arthroplasty populations, lacking assessment of CS, not reporting postoperative outcomes, or focusing solely on surgical technique were excluded, as were case reports, narrative reviews, editorials, conference abstracts, and non-English publications.

Study Selection

All retrieved records were imported into reference management software, and duplicates were removed. Study selection was performed in two stages. First, titles and abstracts were screened to exclude clearly irrelevant studies. Second, full-text articles of potentially eligible studies were reviewed against the predefined inclusion and exclusion criteria. Reasons for exclusion at the full-text stage were documented, and the selection process was summarized using a flow diagram to ensure transparency and reproducibility.

Data Extraction and Quality Appraisal

Data were extracted using a standardized, pre-piloted form, including study design, setting, sample size, patient characteristics, type of arthroplasty, method of CS assessment, outcome measures, follow-up duration, and key findings. Methodological quality was assessed using the Newcastle-Ottawa Scale (NOS), which evaluates selection, comparability, and outcome assessment [11]. Studies were categorized as high, moderate, or low quality based on established thresholds, and quality assessment informed interpretation of the findings and identification of potential sources of bias.

Results

Study Selection

**Figure 1:**
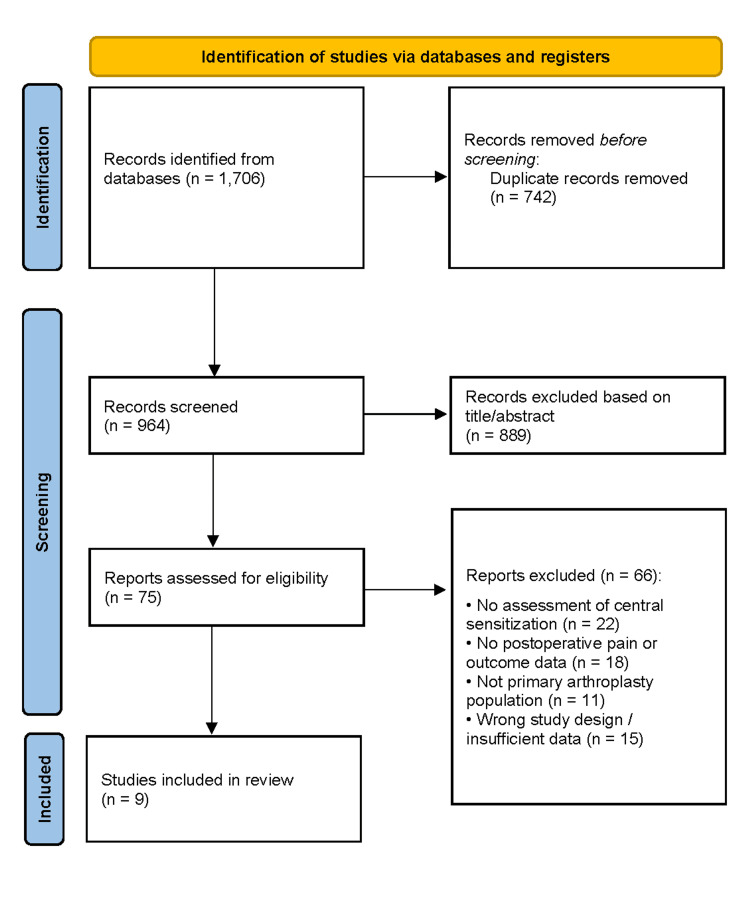
PRISMA flow diagram of study selection PRISMA: Preferred Reporting Items for Systematic Reviews and Meta-Analyses PRISMA flow diagram illustrating the identification, screening, eligibility assessment, and inclusion of studies in the systematic review [10]. The diagram details the number of records identified through database searching and other sources, records screened after removal of duplicates, full-text articles assessed for eligibility, and studies included in the final qualitative and/or quantitative synthesis, with reasons for exclusion at each stage

The database search identified 1,706 records. After removal of duplicates, 964 unique records underwent title and abstract screening, of which 889 were excluded for irrelevance or failure to meet inclusion criteria. Seventy-five full-text articles were assessed, and 66 were excluded due to a lack of CS assessment, absence of relevant postoperative outcomes, inappropriate populations, or insufficient methodological detail. Nine studies met all eligibility criteria and were included in the qualitative synthesis (Figure 1) [1-9].

Study Characteristics

The nine included observational studies comprised more than 1,300 patients undergoing lower-limb arthroplasty. Most were retrospective cohort studies, with three prospective studies, including one multicenter investigation. TKA was the most frequently studied procedure, followed by THA, UKA, and revision TKA. Sample sizes ranged from 66 to 316 participants, with mean ages in the late 60s to early 70s and a predominance of female patients (Table 1).

**Table 1:** Characteristics of the studies included in the systematic review CS: central sensitization; CSI: Central Sensitization Inventory; QST: quantitative sensory testing; PPT: pressure pain threshold; TS: temporal summation; CPM: conditioned pain modulation; PDQ: painDETECT Questionnaire; VAS: visual analog scale; VNRS: Verbal Numeric Rating Scale; WOMAC: Western Ontario and McMaster Universities Osteoarthritis Index; KOOS: Knee injury and Osteoarthritis Outcome Score; BPI: Brief Pain Inventory; FJS: Forgotten Joint Score; PCS-6: pain catastrophizing scale-6; TSK-11: Tampa Scale for Kinesiophobia-11; HADS: Hospital Anxiety and Depression Scale; CPSP: chronic postsurgical pain; PSEQ: Pain Self-Efficacy Questionnaire; MCID: minimal clinically important difference; TKA: total knee arthroplasty; THA: total hip arthroplasty; UKA: unicompartmental knee arthroplasty Summary of studies evaluating the impact of CS on postoperative pain and functional outcomes following lower-limb arthroplasty. CS was primarily assessed using the CSI (25-item or 9-item version, CSI-25, or CSI-9) and, in some studies, combined with QST, including PPT, heat allodynia, TS, and CPM. Neuropathic pain was assessed using the PDQ. Persistent postoperative pain (CPSP) was defined using the VAS or VNRS, while functional and pain outcomes were measured with the WOMAC, KOOS, BPI, FJS, and other patient-reported measures such as PCS-6, TSK-11, HADS, and PSEQ. MCID and patient satisfaction were also reported. Arthroplasty types include TKA, THA, UKA, and revision TKA

Study (author)	Country	Study design	Sample size (n)	Arthroplasty type	Mean age (years)	Sex (% female)	Central sensitization measure	CS cut-off/definition	Comparator	Pain/outcome measures	Follow-up duration	Key results (CS vs non-CS)	Effect size/statistics	Authors’ conclusion
Kim et al. [1]	South Korea	Retrospective cohort	316	Primary total knee arthroplasty (TKA)	72.6 ± 6.5	0.883	Central Sensitization Inventory (CSI) and painDETECT Questionnaire (PDQ)	CS defined as CSI ≥40; neuropathic pain defined as PDQ ≥19	Four groups: CS(+)/NP(+), CS(+)/NP(-), CS(-)/NP(+), CS(-)/NP(-)	WOMAC pain, stiffness, function, total score; walking VAS pain; MCID achievement; analgesic (PCA) consumption; patient satisfaction	2 years	Patients with CS had significantly worse WOMAC pain, function, and total scores preoperatively and at 2 years postoperatively compared with non-CS patients; patients with both CS and neuropathic pain showed the poorest outcomes and lowest MCID achievement	Group comparisons (ANOVA/Kruskal–Wallis); significant intergroup differences for WOMAC subscores and satisfaction (all p < 0.05)	Central sensitization is associated with inferior pain-related patient-reported outcomes after TKA, with cumulative negative effects when combined with neuropathic pain
Sasaki et al. [2]	Japan	Prospective observational study	87	Total knee arthroplasty (TKA)	71.5 ± 5.3	Not reported	Central Sensitization Inventory (CSI)	CS positivity defined by CSI (cut-off not explicitly stated)	CS-positive vs CS-negative; residual CS vs no residual CS	KOOS (TKA); JHEQ (THA); EuroQOL EQ-5D-5L	3 months	Preoperative CS prevalence was high in both groups; residual CS was significantly reduced after surgery. In TKA patients, residual CS was associated with significantly less improvement in KOOS subscales and EQ-5D-5L scores compared with non-CS patients. In THA patients, residual CS had no significant effect on JHEQ subscales or EQ-5D-5L improvement	Regression analysis: EQ-5D-5L negatively correlated with CSI in TKA group (p = 0.017); no correlation in THA group (p = 0.206)	Preoperative and residual central sensitization negatively affect postoperative quality-of-life improvement after TKA, whereas QOL improvement after THA appears independent of residual CS status
Xu et al. [3]	China	Prospective cohort	296	Primary total knee arthroplasty (TKA)	66 (range 49-88)	0.622	Central Sensitization Inventory (CSI-25)	CSis is defined as CSI ≥40	CSI ≥40 vs CSI <40	Brief Pain Inventory (BPI) pain severity, pain interference, total score; MCID achievement	12 months	CS patients had significantly higher postoperative BPI pain severity, interference, and total scores and significantly lower rates of achieving MCID compared with non-CS patients	Anchor-based MCID (total BPI): CS 15.3 vs non-CS 9.8; ROC AUC for total BPI: 0.88 (both groups); MCID achievement significantly lower in the CS group (p < 0.05)	Preoperative central sensitization increases pain-related MCID thresholds and reduces the likelihood of achieving clinically meaningful pain relief after TKA
Kim et al. [4]	South Korea	Retrospective cohort	68	Revision total knee arthroplasty (revision TKA)	69.0 ± 9.5	0.93	Central Sensitization Inventory (CSI-25)	CS is defined as CSI ≥40	CSI ≥40 vs CSI <40	Pain VAS (preop; 3, 6, 12, 24 months), WOMAC pain/function/total, patient satisfaction (new Knee Society Score)	24 months	CS group had significantly higher pain VAS at all postoperative time points and worse WOMAC pain, function, and total scores at 2 years; satisfaction was markedly lower in CS patients compared with non-CS patients	Multivariate logistic regression: CSI ≥40 predicted dissatisfaction (OR 39.08, 95% CI 6.93-220.50, p < 0.001); postoperative 2-year VAS was also predictive (OR 1.86, 95% CI 1.09-3.20, p = 0.024)	Preoperative central sensitization is a strong risk factor for persistent pain and dissatisfaction after revision TKA
Vervullens et al. [5]	Belgium & Netherlands	Prospective multicenter cohort	223	Total knee arthroplasty (TKA)	66.0 ± 7.7	0.498	Central Sensitization Inventory (CSI) and quantitative sensory testing (PPT, heat allodynia, TS, CPM)	CSI ≥40, indicating central sensitization persistence, a disturbed somatosensory profile defined by abnormal CSI/QST at baseline and 1 year	Normal vs recovered vs persistent disturbed somatosensory groups	KOOS pain subscale	12 months	Patients with persistent disturbed somatosensory functioning (including CS) showed significantly less pain improvement and worse KOOS pain scores at 1 year compared with the normal and recovered group	Linear mixed models; significant group × time interactions (p < 0.05)	Persistent centrally disturbed somatosensory functioning, including central sensitization, predicts worse long-term pain outcomes
Kim et al. [6]	South Korea	Prospective observational cohort	91	Total knee arthroplasty (TKA)	70.2 ± 5.7	1	Central Sensitization Inventory (CSI)	High Cis S is defined as CSI ≥40	CSI ≥40 vs CSI <40	VNRS pain at rest and on movement; rescue analgesic (meperidine) consumption; patient satisfaction	3 months	Patients with CSI ≥40 reported significantly higher pain at rest and on movement during postoperative days 1–2 and at 1 and 3 months postoperatively, required higher rescue analgesic doses, and had lower satisfaction regarding pain relief compared with CSI <40 patients	Multivariate logistic regression: CSI ≥40 predicted persistent pain at 3 months (OR 5.091, 95% CI 1.324-19.523, p = 0.016)	High preoperative central sensitization is a strong predictor of severe postoperative pain, increased analgesic requirements, and lower satisfaction after TKA
Kim et al. [7]	South Korea	Retrospective cohort	121	Unicompartmental knee arthroplasty (UKA)	61.2 ± 6.3	0.893	Central Sensitization Inventory (CSI-25)	CS is defined as CSI ≥40	CSI ≥40 vs CSI <40	WOMAC pain, stiffness, function, total score; Forgotten Joint Score (FJS); patient satisfaction (new Knee Society Score)	24 months	CS group demonstrated significantly worse postoperative WOMAC pain, function, and total scores, lower FJS indicating greater joint awareness, and markedly lower satisfaction compared with non-CS patients	Multivariate logistic regression: CSI ≥40 predicted dissatisfaction (OR 6.53, 95% CI 2.30-18.53, p < 0.001)	Preoperative central sensitization is a strong predictor of persistent pain, impaired function, increased joint awareness, and dissatisfaction following UKA
Sonobe et al. [8]	Japan	Retrospective cohort	66	Bilateral total knee arthroplasty (TKA)	72.1 (95% CI 70.7-73.4)	0.773	Central Sensitization Inventory-9 (CSI-9)	High CS is defined as CSI-9 ≥10 points	CS group vs non-CS group	KOOS pain subscale (pre- and postoperative), pain self-efficacy (PSEQ)	3 months	Patients with high CS had significantly worse preoperative and postoperative KOOS pain scores compared with non-CS patients; CS did not influence the magnitude of pain improvement after TKA	Multivariate linear regression: preoperative KOOS pain β = -0.28 (95% CI -18.53 to -0.92, p = 0.031); postoperative KOOS pain β = -0.26 (95% CI -14.09 to -0.44, p = 0.037)	Central sensitization negatively affects pre- and postoperative knee pain in patients undergoing TKA, although overall pain improvement after surgery is preserved
Suzuki et al. [9]	Japan	Retrospective cohort	257	Total hip arthroplasty (THA)	66.9 ± 7.8	1	Central Sensitization Inventory-9 (CSI-9)	CPSP defined as VAS ≥30 mm at 6 months; CS severity categorized using CSI-9	CPSP vs non-CPSP	Motion pain (VAS), CPSP incidence, pain catastrophizing (PCS-6), kinesiophobia (TSK-11), anxiety/depression (HADS), self-efficacy (PSEQ)	6 months	CPSP group showed significantly higher motion pain at 1, 2, and 6 months and a worsening pain trajectory after 1 month compared with non-CPSP patients	Two-way repeated-measures ANOVA/ANCOVA; CPSP incidence 5.1%; age significantly higher in CPSP group (p = 0.01)	A subset of THA patients developed CPSP, characterized by worsening pain trajectories; centrally mediated and psychosocial factors appear to contribute to persistent postoperative pain

CS was primarily assessed using the CSI, either the 25-item version or the abbreviated CSI-9, most commonly using a threshold score of 40 to define CS. One study incorporated neuropathic pain screening, and one multicenter study combined CSI scores with QST to characterize somatosensory function.

Pain-related outcomes were evaluated using validated measures such as the visual analog scale (VAS), Verbal Numeric Rating Scale (VNRS), Western Ontario and McMaster Universities Osteoarthritis Index (WOMAC), Knee Injury and Osteoarthritis Outcome Score (KOOS), and Brief Pain Inventory (BPI). Follow-up durations ranged from three to 24 months.

Methodological Quality

Methodological quality, assessed using the NOS, was moderate to high across studies, with scores ranging from 6 to 9. Seven studies were rated as high quality, and one was rated as moderate quality. Most studies demonstrated strong cohort selection, appropriate outcome assessment, adequate follow-up, and reasonable control for key confounders, indicating a low to moderate risk of bias overall (Table 2).

**Table 2:** Methodological quality assessment of the included studies NOS: Newcastle-Ottawa Scale Methodological quality of included studies was assessed using the NOS [11]. The NOS evaluates observational studies across three domains: selection (0-4 points), which assesses the adequacy of cohort selection, representativeness, and ascertainment of exposure; comparability (0-2 points), which evaluates control for confounding factors; and outcome (0-3 points), which assesses the adequacy of outcome assessment, follow-up duration, and completeness. Total scores range from 0 to 9, with higher scores indicating better methodological quality. Quality ratings were categorized as high (7-9 points) or moderate (5-6 points) based on the total score

Study (author)	Selection (0-4)	Comparability (0-2)	Outcome (0-3)	Total score (0-9)	Quality rating
Kim et al. [1]	4	2	3	9	High
Sasaki et al. [2]	3	2	2	7	High
Xu et al. [3]	4	2	3	9	High
Kim et al. [4]	4	2	3	9	High
Vervullens et al. [5]	4	2	3	9	High
Kim et al. [6]	3	2	2	7	High
Kim et al. [7]	3	2	2	7	High
Sonobe et al. [8]	3	2	2	7	High
Suzuki et al. [9]	3	1	2	6	Moderate

Pain Outcomes and CS

Across all studies, preoperative CS was consistently associated with higher postoperative pain intensity and an increased risk of persistent or chronic postoperative pain. Patients with CS showed less favorable pain trajectories, including incomplete pain resolution or secondary worsening over time, compared with patients without CS [6-9]. CS remained an independent predictor of residual pain after adjustment for demographic and clinical factors in several cohorts [6,8].

Somatosensory Dysfunction and Pain Trajectories

Studies incorporating somatosensory profiling demonstrated that patients with persistent disturbed somatosensory function, particularly those characterized by CS, experienced less improvement in pain over time and worse long-term pain outcomes. In contrast, patients with normal or recovered somatosensory function showed sustained postoperative pain improvement, suggesting that centrally mediated pain mechanisms limit postoperative recovery in a subset of patients [5].

Functional Outcomes, Satisfaction, and Quality of Life

CS was also associated with poorer functional outcomes, lower patient satisfaction, and reduced quality of life after arthroplasty. Patients with CS, particularly when combined with neuropathic pain features, reported worse functional scores, lower rates of achieving minimal clinically important differences, greater joint awareness, and higher dissatisfaction following primary and revision arthroplasty [1,4,7]. Quality-of-life measures were adversely affected by residual CS after surgery, particularly in TKA populations [2,3].

Discussion

This systematic review demonstrates that preoperative CS is consistently associated with less favorable pain-related outcomes following hip and knee arthroplasty [1,3-7]. Across a range of procedures and follow-up durations, patients exhibiting features of CS experienced higher postoperative pain intensity, an increased likelihood of persistent pain, reduced functional improvement, greater analgesic requirements, and lower satisfaction compared with patients without evidence of altered central pain processing [1,3-8]. These findings suggest that pain persistence after arthroplasty is not solely driven by peripheral joint pathology or surgical factors [4,7,9].

The observed association between CS and poorer outcomes highlights the importance of pain processing mechanisms in shaping postoperative recovery [7-9]. Even when surgical reconstruction is technically successful, heightened central nervous system responsiveness may limit the expected benefits of joint replacement by amplifying pain perception and impairing pain modulation [8,9]. This may explain the variability in patient-reported outcomes that is frequently observed in clinical practice despite similar radiographic and surgical results [3,4,7].

From a clinical perspective, the findings support the potential value of incorporating CS assessment into routine preoperative evaluation [5,6]. Identifying patients with centrally mediated pain characteristics may allow for more realistic expectation setting, targeted perioperative pain management strategies, and individualized rehabilitation approaches [1,3,7]. Such an approach could help optimize outcomes by addressing both peripheral and central contributors to pain [7-9].

Limitations

Despite the overall consistency of findings, this review has several limitations. Substantial heterogeneity was present across the included studies in the definition and assessment of CS, the instruments and thresholds used to classify patients, the outcome measures reported, and the duration of follow-up. This variability limits direct comparison between studies and precludes quantitative synthesis. In addition, most included studies were observational in design, which increases susceptibility to residual confounding and limits causal inference. Differences in patient populations, surgical procedures, and perioperative pain management protocols may have further influenced outcomes. Finally, CS was assessed primarily at a single preoperative time point, providing limited insight into postoperative changes or the potential reversibility of centrally mediated pain mechanisms. Future studies should adopt standardized assessment frameworks, incorporate longitudinal evaluation of CS, and investigate whether targeted perioperative interventions can modify CS-related risk and improve postoperative outcomes.

## Conclusions

This systematic review provides robust evidence that CS is a key determinant of persistent pain, functional impairment, reduced patient satisfaction, and diminished quality of life following hip and knee arthroplasty. The findings highlight that unfavorable postoperative outcomes can occur despite technically successful surgery when centrally mediated pain mechanisms are present. Recognizing CS as part of the preoperative assessment may improve risk stratification and support more individualized perioperative management strategies aimed at optimizing pain relief and functional recovery after joint replacement.
